# The Evolving Landscape of Male Varicocele Pathophysiology in the Era of Multi-Omics: A Narrative Review of the Current Literature

**DOI:** 10.3390/biology13020080

**Published:** 2024-01-28

**Authors:** Carlos Munoz-Lopez, Anne Wong, Kieran Lewis, Raevti Bole, Sarah C. Vij, Scott D. Lundy

**Affiliations:** 1 Glickman Urological and Kidney Institute, Cleveland Clinic, Cleveland, OH 44195, USA; 2 Cleveland Clinic Lerner College of Medicine, Cleveland, OH 44195, USA

**Keywords:** varicocele, spermatogenesis, male infertility, sperm

## Abstract

**Simple Summary:**

Male-factor infertility is a condition affecting nearly half of all infertile couples, and the most common correctable cause is a cluster of dilated veins above the testicle called a varicocele. How a varicocele forms and how exactly it impairs sperm production remains a mystery. In this review article, we explore the historical studies describing various theories for this condition. We also review the modern literature that uses large data sets, termed “-omics” analyses, to better understand the genes, RNA, proteins, and bacteria that may play a role. Using this information, future non-surgical treatments for varicoceles may be on the horizon for the millions of men with this condition.

**Abstract:**

Male-factor infertility is implicated in over half of the millions of cases of infertility worldwide, and varicoceles are the most common correctable cause of male-factor infertility. The pathophysiologic mechanism for varicoceles is complex and next-generation technologies offer promising insights into the molecular underpinnings of this condition. In this narrative review, we highlight historical and contemporary paradigms associated with varicoceles, with an emphasis on the biological underpinnings of this disease. Specifically, we review the literature describing the underlying causes of varicoceles, discuss the molecular and cellular mechanisms causing pathological changes in some (but not all) men, and highlight key articles regarding the next-generation analyses (e.g., transcriptome, epigenome, proteome, and microbiome) being applied to better understand the condition and its treatment. These data demonstrate an ongoing evolution of the knowledge of varicoceles and the potential for improved personalized care in the future for men with this condition.

## 1. Introduction

Nearly half of the fifty-five million couples suffering from infertility worldwide will have difficulty conceiving due to male-factor infertility [1,2]. Varicocele, defined as an abnormal enlargement of the pampiniform plexus of gonadal veins above the testicle, is the leading correctible cause of male-factor infertility and is found in 35–44% of men with primary infertility and 45–81% of men with secondary infertility [3,4,5,6]. Clinically, varicoceles reduce sperm quantity and quality, slow testicular development in adolescents, cause dull discomfort and pain, and decrease testosterone levels [7,8,9,10]. While prior studies have identified numerous anatomic, genetic, and molecular contributors to varicoceles, the true factors underlying the development of male varicoceles remain largely unknown [11]. Furthermore, the molecular underpinnings predisposing only a subset of men to pathological changes continue to evolve and haveincreasing relevance for clinical management [12]. 

The next-generation omics advances (proteomics, transcriptomics, epigenomics, etc.) have begun to provide insight into understanding the pathophysiology of infertility associated with varicoceles [13]. These studies have identified numerous molecular changes in men with varicoceles, including regional hypomethylation or reduced expression of spermatogenesis-related proteins, which may reduce sperm viability and negatively impact fertility [14]. While thought-provoking, these studies remain limited by a relatively low number of subjects, heterogeneity between studies, and limited validation in secondary cohorts. Moreover, there remains a lack of demonstrated causality between omic changes and infertility, as well as limited data on how these changes impact patient management. 

In this narrative review, we outline modern data exploring varicocele-associated male-factor infertility in the era of big data. We discuss the cellular and molecular changes seen in male-factor infertility and their corresponding association with the treatment of varicoceles. Lastly, we describe novel next-generation approaches to basic science investigation regarding varicoceles and strategies to inform causality in this disease process.

## 2. Materials and Methods 

**Figure 1:**
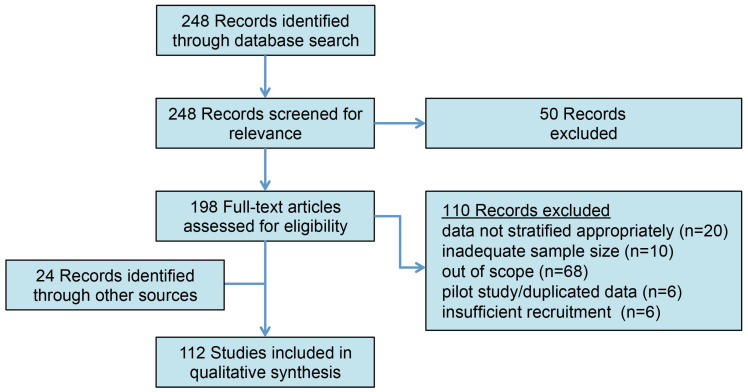
The PRISMA outline of article selection. The initial search identified 248 records for review. Following the abstract review and the exclusion of non-relevant studies, 112 studies were included in the qualitative analysis.

A comprehensive literature search for articles with no specified publication date range was conducted according to the PRISMA guidelines on 1 November 2023 using the PubMed database for the following MeSH terms: varicocele (OR varicoceles) AND infertility (OR infertile, OR subfertility), AND genome (OR genomic, OR proteome, OR proteomics, OR protein, OR ROS, OR “reactive oxygen species”, OR epigenome, OR transcriptome, OR molecular) (Figure 1). The abstracts were screened, and full-length articles were reviewed and included if they were written in English and if their titles/abstracts were relevant to the context of the review. Commentaries, editorials, and non-English articles were omitted. Additional relevant articles and historical references were manually identified from the reference lists of included articles. 

## 3. Results

### 3.1. Anatomic Etiology of Varicoceles

Varicoceles are generally left-sided due to the elevated position of the left kidney causing increased hydrostatic pressure. Some clinicians have instead attributed this laterality bias to the 90-degree angle between the insertion of the gonadal vein to the left renal vein, but this is in direct contradiction to the fundamental hydrostatics principle of Pascal’s law and the accompanying visual experiment of Pascal’s vases [15,16]. Alternatively, others have proposed that skeletal growth leads to the compression of the left renal vein from the superior mesenteric artery and the aorta, thereby contributing to the increased hydrostatic pressure on the left side. However, it is important to distinguish this theorized mechanism from the Nutcracker Syndrome, as it is unknown whether there is a shared venous congestion pathway between these two conditions [17,18]. Genetics also appear to play a strong role, with an increased varicocele prevalence in men with close relatives also having the condition. Finally, some have proposed that the internal spermatic veins lack functional valves, resulting in pooling and congestion [19]. This hypothesis is heightened by the positive association between varicose veins and varicoceles: a population-based study found that patients with varicoceles were nearly fivefold more likely to have varicose veins [20]. While varicoceles are commonly diagnosed as a left-sided disease, bilateral varicoceles are also quite common. Even unilateral varicoceles, however, appear to have bilateral testicular dysfunction through unknown mechanisms. The etiology of varicoceles remains complex and further work will be necessary to better define the interplay between these anatomic, environmental, and biological factors. 

### 3.2. Pathophysiology of Varicocele-Related Testicular Tissue Dysfunction

**Figure 2:**
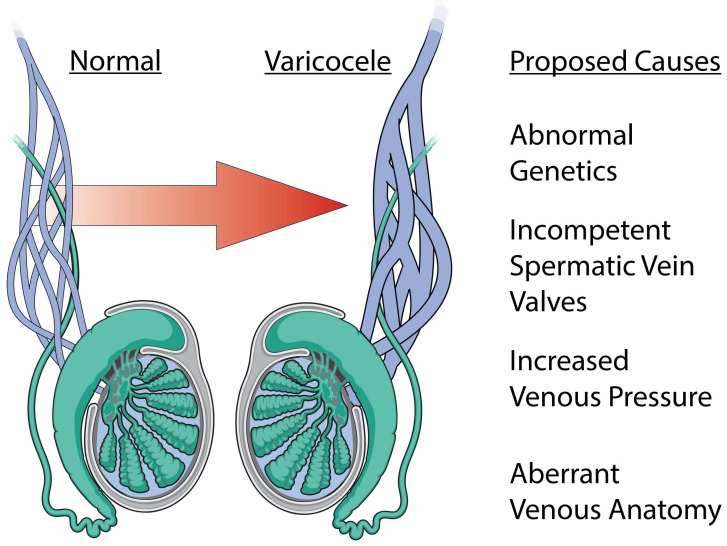
Etiology of varicocele formation. A varicocele is a dilation of the male pampiniform plexus and has a myriad of proposed etiologies. The most common proposed causes include abnormal genetics, incompetent spermatic vein valves, increased venous pressure, and aberrant venous anatomy.

**Figure 3:**
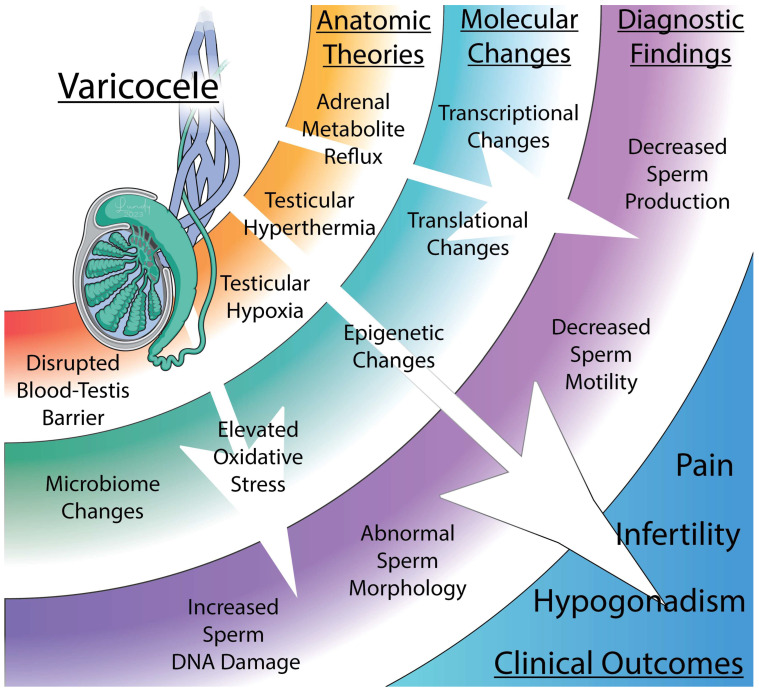
The anatomic, molecular, and diagnostic findings associated with varicoceles. Varicocele formation is thought to negatively affect male fertility through a variety of mechanisms. Dilation of the pampiniform plexus may increase adrenal metabolite reflux, induce testicular hyperthermia or hypoxia, or disrupt the blood–testis barrier. These anatomic changes affect protein expression and change the native microbiome which can result in decreased sperm motility, abnormal morphology, and increased sperm DNA damage. Collectively, these factors lead to several clinical outcomes, including infertility, hypogonadism, and testicular pain.

Regardless of how a varicocele anatomically forms, the mechanisms impairing testicular and cellular function have been thoroughly studied but remain controversial (Figure 2 and Figure 3). Furthermore, the reason why these changes occur only in a subset of men with varicoceles remains enigmatic. Here, we summarize the proposed mechanisms and the strength of evidence supporting them. 

#### 3.2.1. Oxidative Stress

Oxidative stress (OS) has been a well-studied potential mechanism associated with varicocele-induced male-factor infertility. OS refers to elevated levels of free radicals (e.g., O_2_^−^ and NO_3_^−^), which cause direct cellular and DNA damage. While some degree of OS is essential for certain physiological processes, the human body normally has protective antioxidant mechanisms in place to maintain this delicate homeostasis. When reactive oxygen species (ROS) surpass these levels, there is a corresponding decrease in sperm production and function [21]. Multiple studies have suggested that infertile males with grade II/III varicoceles have elevated levels of ROS and nitric oxide (NO), compared to the age-matched controls without varicoceles [22,23]. This increased OS can lead to sperm membrane damage, DNA damage, impaired spermatogenesis, and poor sperm motility [24,25,26,27]. Tempering this enthusiasm, however, are the clear data suggesting that these associations only affect a subset of infertile men with or without varicoceles. Nevertheless, elevated OS may be clinically used to further justify varicocelectomy in men with infertility.

#### 3.2.2. Reflux of Metabolites

Increased levels of renal and adrenal metabolite reflux to the testicle have been proposed to alter the testis microenvironment, resulting in Leydig and Sertoli cell injury [28,29]. Specifically, catecholamine reflux (primarily norepinephrine) has been shown to increase testicular vasoconstriction and induce hypoxia, leading to downstream metabolic and developmental changes [30]. Serotonin and NO levels have also been shown to be elevated in the spermatic veins of varicocele patients [31,32]. Increased serotonin production may also lead to increased angiogenesis, which may promote the distribution of toxic metabolites throughout the testis. Despite this compelling hypothesis, the experimental data regarding this are very limited and has primarily been restricted to animal studies. Furthermore, several studies have failed to show an association between the presence of clinical varicoceles and a reflux of metabolites [33,34]. Future research should aim to better characterize the clinical and mechanistic relevance of these findings [35].

#### 3.2.3. Testicular Hypoxia

Another potential mechanism for varicocele-induced infertility is testicular hypoxia, resulting from venous stasis and decreased flow. Multiple studies have demonstrated that hypoxia-inducible factor 1-alpha (HIF-1α), a well-known marker of hypoxia, is upregulated in males with varicoceles [36,37,38]. The expression of HIF-1α mediates the expression of several key genes including p53, VEGF, GLUT, Bax, and Caspase-3. These genes have been implicated in DNA fragmentation and sperm apoptosis [36,39,40,41]. Interestingly, varicocele ligation has been associated with downregulation of these genes [42]. These data suggest upregulated gene expression in the presence of varicoceles, and further studies with novel assays to measure hypoxia, including next-generation imaging modalities, may prove to be useful [43]. 

#### 3.2.4. Temperature Regulation

Temperature regulation within the testes can also be affected by a varicocele. Dysfunction in spermatic venous drainage has been shown to increase testicular temperature, resulting in ROS production and impaired sperm production [21]. The normal scrotal temperature is approximately 34 °C (about 2.5 °C below body temperature) to facilitate spermatogenesis [44]. Sperm concentration has been shown to decrease by 40% as scrotal temperature increases by 1 °C, reflecting the importance of scrotal temperature regulation [45,46]. Patients with varicoceles have been found to have elevated scrotal temperatures (ranging from 0.2 to 0.6 °C) compared to controls [47]. Studies have also demonstrated improved thermoregulation with varicocelectomy, suggesting that this process may be reversible [48,49]. Despite this, the association between increased scrotal temperature and impaired fertility remains unclear, as not all men with increased scrotal temperature are infertile [50]. This concept is demonstrated well in the manuscript by Garolla and colleagues, who conducted a case–control study to evaluate circadian temperature variability amongst men with varicoceles compared to healthy controls. The data demonstrated that men generally experience variability in intratesticular temperature throughout a 24-hour period, but certain conditions (e.g., obesity or higher varicocele grade) were more consistently associated with elevated temperature and minimal temperature variability. Interestingly, however, the authors also reported a difference in temperature between the affected and unaffected testicles, suggesting that hyperthermia may not always have a global increase in scrotal temperature [51]. 

Despite all of this work, none of the aforementioned hypotheses fully explain the disease process, as none can fully characterize pathogenic varicoceles from incidental varicoceles nor predict who would benefit from repair. 

### 3.3. Cellular Consequences of Varicoceles

#### 3.3.1. Cellular Effects

Male varicoceles have been shown to negatively affect both Sertoli and Leydig cell functions, causing decreased sperm production. Histologic studies on testicular biopsies of men with varicoceles have demonstrated that patients with varicoceles have a heterogeneous pattern of spermatogenic arrest at the spermatid or primary spermatocyte stage [52,53]. Varicoceles may also lead to abnormal expression of sperm proteins, leading to changes in spermatid differentiation, sperm acrosome function, and abnormalities in the nucleus [54,55,56]. Animal models have found that varicoceles are associated with reduced numbers of Leydig cells [57,58,59], which compromises the intratesticular testosterone production necessary for sperm maturation. Indeed, it has been shown that the testicular microenvironment in men with varicoceles negatively affects the germ cell function through this and multiple other mechanisms [60]. Specifically, one study by Fang and colleagues found that the presence of varicoceles was associated with a proinflammatory state (e.g., increased expression of interleukin-1 and tumor necrosis factor alpha), and the authors hypothesized that this proinflammatory state was associated with germ cell dysfunction [61]. In summary, the literature currently suggests that varicoceles are associated with heterogeneous Sertoli and Leydig cell dysfunction, reduced numbers of Sertoli or Leydig cells, and upregulation of pro-inflammatory factors. 

#### 3.3.2. Sperm DNA Fragmentation and Clinical Outcomes

Sperm DNA fragmentation (SDF) is a major clinical finding in patients who experience varicocele-associated infertility, and the current AUA and European guidelines recommend evaluation of SDF in couples with recurrent pregnancy loss or other specific circumstances [62,63]. SDF, including single/double-stranded DNA breaks, is associated with oxidative stress and increased presence of ROS [33,64,65]. In a recent meta-analysis including 845 varicocele patients and 2377 healthy controls, patients with varicoceles were found to have higher rates of SDF compared to controls [65]. SDF may negatively affect pregnancy outcomes, including recurrent pregnancy losses, in both human and animal studies [66,67,68]. These data highlight that beyond negatively affecting sperm quantity, varicocele may result in changes that negatively affect fertilization even in the setting of assisted reproduction. However, there may be some degree of reversibility with this process as a meta-analysis of nearly 300 patients demonstrated that varicocelectomy was associated with reduced markers of SDF [69]. 

#### 3.3.3. Genetics and Varicoceles

Numerous studies have demonstrated an association between male infertility and genomic abnormalities. One of the first evolutions in this field was the association between Y-chromosome microdeletions and male-factor infertility [70,71]. In the early 2010s, several single-nucleotide polymorphisms (SNPs) were found to be associated with varicoceles, including mutations in glutathione S-transferase, polymerase gamma, and methylenetetrahydrofolate reductase [72,73,74,75]. Other studies have also identified mutations that are associated with increased oxidative phosphorylation and OS [76]. Whole-exome sequencing in a cohort of patients with varicoceles has identified multiple genetic variants associated with varicoceles (e.g., AAMP, SPINT1, or MK167) [77]. A recent study of three men with varicoceles performed next-generation RNA sequencing to explore preferentially upregulated genes in men with varicoceles and identified cystic fibrosis transmembrane conductance regulator (CFTR), suggesting a potential role of this protein in the pathogenesis of varicoceles [78]. Although intriguing, this study has not been independently validated and additional studies will be necessary to further characterize the phenotypes of these select gene mutations in a more robust cohort of patients. 

#### 3.3.4. Blood–Testis Barrier

The blood–testis barrier (BTB) is one of the tightest blood–tissue barriers in the human body and divides the seminiferous tubule into distinct basal and apical compartments. The basal compartment is responsible for spermatogonia stem cell renewal and early differentiation, while the apical compartment protects haploid cells from immune recognition. In a study by Oh and colleagues, animals with varicoceles were found to have upregulated proinflammatory proteins compared to those without varicoceles [79]. The authors concluded that the increase in pro-inflammatory cytokines may mediate disruption of the BTB, thereby facilitating immune-mediated damage of Sertoli cells. Other studies have observed similar disruption of the BTB through the dysregulation of tight junctions. For example, the downregulation of claudin-11 mRNA, an important protein for tight junction formation, was observed in varicocele-affected rat models [80,81]. These studies also demonstrated an increased expression of the TGF-β transcript in rats with varicoceles, which has been shown to loosen tight junctions at the BTB. These findings collectively indicate that varicoceles may cause disruption of the BTB by inducing inflammatory state. Further work to explore whether the stage of varicocele development is directly associated with a heightened inflammatory state may be beneficial, as this could affect the management of such patients. 

### 3.4. Next Generation Technologies

#### 3.4.1. Epigenome

The epigenome refers to the combined post-translational genetic modifications that do not alter the underlying genetic code, but rather its degree of utilization and accessibility [82]. Alterations in the epigenome of spermatozoa and its precursors are seen in men with varicoceles, and it has been postulated that these alterations may contribute to male-factor infertility. For example, global hypomethylation has been observed in men with varicoceles, and hypomethylation predisposes DNA to damage, independently of other exacerbating factors (e.g., ROS production) [75,83]. In addition to global hypomethylation, differences in regional methylation patterns have been observed in patients with varicoceles. In a comparison of twenty-six controls and twenty-six men with varicoceles, fifty-nine differentially methylated CpG sites were seen, with the majority being hypomethylated in the varicocele group [75,83]. Various research efforts have focused on determining the underlying reasons for the hypomethylation observed in these patients. In a study by Rashidi and colleagues, the expression of DNMT3A and DNMT3B was elevated in 35 men with varicoceles at both the mRNA and protein levels. These enzymes function as both methyltransferases and dehydroxymethylases, depending on the cellular environment, and their increased expression could contribute to global hypomethylation [84]. In a varicocele-induced rat model there was an increased expression of TET2 mRNA, a key regulatory protein in the demethylation pathway, and this overexpression may have contributed to the global hypomethylation observed in rats with varicoceles [85]. Collectively, these data provide critical insights into the potential mechanistic role of the sperm epigenome. Further research will be required to fully evaluate these data and their clinical consequences, including how such data may affect diagnosis and treatment paradigms.

#### 3.4.2. Transcriptome

Gene expression is generally repressed in spermatozoa due to spermatic DNA being highly packaged and condensed, making a study of the sperm transcriptome challenging. Much of the RNA present in mature sperm is thought to be remnants from spermatogenesis, and the true transcriptional activity of spermatozoa is poorly defined [86]. With this limitation in mind, some studies have evaluated the associations of varicoceles with the sperm transcriptome and have identified differences in gene expression in subjects with varicoceles [80,81,87]. However, there are few data regarding how these aberrantly expressed proteins directly affect male fertility. One study demonstrated the downregulation of the heat shock protein family A2 (HSPA2) mRNA, which plays an integral role in regulating egg–sperm recognition [88]. Alternatively, other studies suggested dysregulation in key proteins that mediate sperm development and stability of sperm-stored DNA. For example, TRPV1 mRNA expression, a cation channel that plays a role in spermiogenesis, was found to have significantly lower expression in men with varicoceles [89]. Similarly, elevated protamine-1/protamine-2 ratios have also been observed in varicoceles, which is thought to have downstream consequences impacting chromatin packaging, ultimately affecting the expression of key genes [87]. 

Animal models have proven to be valuable in the study of the male transcriptome. One study used a varicocele model to evaluate the effect of resveratrol, a naturally occurring polyphenol, and discovered decreased protein expression of protamine I/II and HSPA2 [90]. These results indirectly suggest that the downregulation of these proteins contributes to the pathogenesis of male-factor infertility. Other data implicate structural abnormalities in sperm resulting in poor sperm function. CatSper is a Ca^2+^ channel expressed in sperm that functions to facilitate motility by allowing the influx of Ca^2+^. A rat model demonstrated reduced CatSper gene expression after the induction of varicoceles [80]. To summarize, the transcriptome profile of those with varicoceles differs from those without it, particularly in important fertility-related genes, though how each difference contributes to infertility is difficult to discern. These data provide evidence for the mechanistic relevance of the male transcriptome and further work will be needed to characterize this evolving field.

#### 3.4.3. Proteome

In addition to the epigenome and transcriptome, the male proteome has been of interest in the varicocele literature. In 2013, a key paper by Del Giudice and colleagues found that varicocelectomy was associated with changes in the male seminal plasma protein profile, suggesting that oxidative stress induced by varicoceles may change the proteomic landscape and contribute to male-factor infertility [91]. Since this study, several proteins (e.g., AKAP, CABYR, SEMG1, APOPA1, ACR, RSPH1, SPA17, RSPH9, and DNAH17) have been implicated in male-factor infertility. These proteins are associated with a variety of functions including motility, morphology, and mitochondrial fitness [1,2,24]. Other studies have reported that patient behavior may affect protein expression. For example, some have reported that smoking is associated with the underexpression of several key proteins [92]. Finelli and colleagues reported that patients with varicoceles differentially expressed several key proteins (e.g., fatty acid synthase, myeloperoxidase, or mitochondrial aconitate hydratase) specifically associated with DNA repair [93]. Other studies have also evaluated the impact of varicocelectomy and found that some of these aberrant proteins normalize after varicocelectomy [94,95]. 

Mitochondrial proteins have also garnered attention. Mitochondrial proteins broadly function primarily as antioxidants and it is hypothesized that the underexpression of these proteins may lead to increased oxidative damage [96]. Some groups have suggested that these aberrantly expressed proteins may serve the role of biomarkers for oxidative stress. However, further research is warranted to evaluate this role.

#### 3.4.4. Microbiome

The microbiome is a term describing the sum of bacteria, viruses, and fungi living within a niche in the human body. Recent work has identified a key role for the microbiome in numerous clinical and urological conditions. Traditionally, the role of the microbiome in male infertility has been viewed through the lens of infections from pathogenic bacterial overgrowth, and the relationship between bacterial orchitis and male-factor infertility has been well documented [97,98]. Single pathologic species such as *Ureaplasma* are known to cause semen parameter changes in a subset of infertile men through unknown mechanisms [99,100]. This relationship also extends to infertile men with varicoceles, as it has been shown that infertile men with varicoceles are more likely to be colonized by *Ureaplasma urealyticum* than infertile men without varicoceles [101,102]. 

New research into the role of the gut microbiome and how it relates to human diseases has greatly expanded in recent years [103,104]. Advances in technologies such as marker-based 16S rRNA sequencing have allowed microbiome research to expand beyond traditional culture techniques to explore microorganism diversity in the semen and testicular environments, where bacterial quantity is far lower than in other organs such as the gut [105]. Collectively, these studies demonstrate that men with infertility have distinct microbiota from healthy men, although casual mechanisms implicating specific organisms have yet to be elucidated [106,107,108]. A recent study by our group in 2021 evaluated gut, semen, and urine microbiomes in both fertile and infertile men utilizing both 16S rRNA sequencing and shotgun metagenomics. This work showed a distinct microbiome in infertile men in all three locations, with numerous pathogenic taxa overrepresented and appearing to correlate with decreased concentration, motility, and morphology. Subgroup analyses specifically evaluating the semen of infertile men with varicoceles found that anaerobic organisms, including *Bacteroides* and *Peptoniphilus* species, were highly overexpressed [109]. This work raises an intriguing hypothesis that a shift to more anaerobic seminal microbiota in men with varicoceles may impact fertility and may be a newly proposed theory to explain the decreased sperm concentration and elevated oxidative stress seen in those with varicoceles. The field of microbiota research is rapidly expanding, and continued research and technological advances will likely yield new insights into the role of the microbiome in male infertility and varicocele.

### 3.5. Molecular Effects of Varicocele Treatment

The current AUA guidelines recommend surgical ligation of varicoceles in infertile men with clinical (e.g., palpable) varicoceles and abnormal semen parameters [63]. Varicocelectomy clearly improves semen parameters and pregnancy outcomes [79,110]. In addition to being an effective treatment option for male-factor infertility, varicocele repair may also allow for targeted evaluation of the testicular environment before and after the treatment to identify causal aspects of testicular dysfunction. 

One of the first hypothesis-generating studies in this space was conducted by Camargo and colleagues who evaluated 18 men with semen studies before/after varicocelectomy. Post-varicocelectomy samples were found to have significant changes in proteomic profile following repair. Notably, patients with varicoceles were found to have enriched expression of stress-response proteins, while the treatment arm had a microenvironment more consistent with the normal testicular microenvironment [111]. This study benefitted from having patients serve as their own internal control; however, it was limited due to the low number of patients and restricted generalizability. Another study evaluated sperm mitochondrial DNA (mtDNA) copy number in 14 men before and after varicocelectomy. Sperm mtDNA copy number is inversely correlated with fertility and mtDNA is continuously shed throughout spermiogenesis. The analysis found a significant reduction in mtDNA copy number after the surgery and an inverse correlation between sperm motility and mtDNA copy number [112], suggesting that the sperm microenvironment in the presence of varicoceles may produce more functionally robust sperm. 

Next-generation sequencing of testicular tissue has been evaluated as a potential predictor of who will respond to varicocelectomy. In a 2017 study published in the *Journal of Urology*, transcriptome analysis was performed on testicular biopsies from eighty-tree infertile men undergoing varicocelectomy, and transcription profiles were compared between those who responded to varicocele repair versus those who did not. The study found significant upregulation of cell cycle-related genes and downregulation of antioxidant genes, providing potential biomarkers for which patients will respond to the repair [41]. However, the technology and methodology proposed in this study have not been validated in secondary cohorts. Overall, these data suggest that varicocele occurrence is associated with changes in the testicular microenvironment, but such changes are potentially reversible with varicocelectomy.

## 4. Conclusions

Varicocele pathophysiology and its ensuing male-factor infertility remains a complex disease process with an evolving knowledge base. While historic paradigms suggest that varicocele is strictly an anatomic malformation, we now understand that the condition results in alterations in nearly every major aspect of molecular machinery in the testicle. Further research will be needed to better illustrate how these molecular mechanisms may affect treatment options for men with varicocele-associated infertility and help rationally inform future treatment strategies.

## Data Availability

Not applicable.

## Abbreviations

AUA	American Urologic Association
BTB	Blood–testis barrier
DNA	deoxyribonucleic acid
HIF-1α	Hypoxia-inducible factor 1-alpha
HSPA2	Heat shock protein family-A2
mtDNA	Mitochondrial DNA
NO	Nitric oxide
OS	Oxidative stress
RNA	Ribonucleic acid
ROS	Reactive oxygen species
SDF	Sperm DNA fragmentation
SNP	Single nucleotide polymorphism
